# Supplementary material for: Comparative Metabolomics Analysis Reveals Sterols and Sphingolipids Play a Role in Cotton Fiber Cell Initiation
Source: Int J Mol Sci. 2021 Oct 23;22(21):11438. doi: 10.3390/ijms222111438 (PMC8583818; doi:10.3390/ijms222111438)
Supplement: Supplementary file 1 [file ijms-22-11438-s001.zip › Table S1.pdf]

Table S1. The sequence of primer used to detect expression level in RT-qPCR

gene	primer	sequence(5'-3')
GhGCS2680	GCS2680-1	GGCACCTAAGCTCTCACTTG
	GCS2680-2	CTTTGGGAGTGGCTGCTTTC
GhGCS7060	GCS7060-1	TATCTCTGCCTTCCGATCTC
	GCS7060-2	TAATCCCACCACCAACCAAC
GhGCS2810	GCS2810-1	TTGTCTGTGTTTGGTCGATC
	GCS2810-2	CCGTGGTAGATTGACCTGAT
GhGCS7230	GCS7230-1	GGCACCTAAGCTCTCACTTG
	GCS7230-2	CCTCTCTCTGCTAAACCTCG
GhIPCS1	IPCS1-1	CGCTTTACATTGGTCGCGAAGC
	IPCS1-2	GAACTCCTCGAGCAGCTACACC
GhIPCS2	IPCS2-1	TTGGTGCAGGGTGCTAGCATTT
	IPCS2-2	CTAGCACACTCTCCGGTTTCGG
GhIPCS7	IPCS7-1	CCGAGAGGGTTCTAAGCTTGCC
	IPCS7-2	GTGAGCACAAAGACCAGGGTGA
GhIPCS8	IPCS8-1	ATGGTCTAGCTGCCCATGGAGT
	IPCS8-2	AATGCTAGCACCCTGCACCAAA
GhCYP710A1	CYP710A1-1	GAAGCACCGAACTTTCCCACT
	CYP710A1-2	GAGTAGCACAATCACCAAGGG
GhCYP710A2	CYP710A2-1	CACCTTATCTTCGCTAACGTC
	CYP710A2-2	AGCACAATCACCAAGGGTTTC
GhPSAT1	PSAT1-1	TTGAGGAGCGAGACCTTTAC
	PSAT1-2	TGAACCAAGAAGAGGAGCTC
GhHMGR1	HMGR1-1	GGATGTCATTGGCATCTCTGG
	HMGR1-2	TGATGCAATGCGAGCTCTCGA
GhCPI1	CPI1-1	GAACGATGTACCACACACGAC
	CPI1-2	GGGTTTCTCGTCAATCCTGAG
GhSMT1	SMT1-1	GGTCTTCCGGATATCAGGTTG
	SMT1-2	ACTTCCCTTGGGAGCTAATCC
GhSMT2	SMT2-1	CGATCAACGATTACCAAGTG
	SMT2-2	AGTAACAAGAGGTGGAACACA
GhDWF1	DWF1-1	GCTCAAGGCTACTCAAGGTG
	DWF1-2	CTCCGACACTGCATACTGTG
GhHIS3	HIS3-up	GAAGCCTCATCGATACCGTC
	HIS3-dn	CTACCACTACCATCATGGC
